# Thoracic Outlet Syndrome Presenting With Unilateral Upper Extremity Venous Congestion: A Diagnostic Role for Dermatology

**DOI:** 10.7759/cureus.104409

**Published:** 2026-02-27

**Authors:** Alyssa Sayegh, Lauren Fleshner, Frederick Pereira, Mehmet Fatih Atak, Banu Farabi

**Affiliations:** 1 Dermatology, New York Medical College, Valhalla, USA; 2 Dermatology, Mount Sinai Hospital, New York, USA; 3 Dermatology, New York Medical College/NYC Health + Hospitals/Metropolitan, New York, USA

**Keywords:** cutaneous vascular changes, dermatologic diagnosis, skin manifestations of systemic diseases, thoracic outlet syndrome, venous congestion, venous thoracic outlet syndrome

## Abstract

Thoracic outlet syndrome (TOS) is a constellation of symptoms associated with compression of the neurovascular bundle of the brachial plexus or subclavian vessels. TOS can be classified as neurogenic, arterial, or venous depending on the structure that is compressed. Symptoms include arm pain, swelling, fatigue, paresthesia, weakness, and discoloration of the hand. While neurogenic and vascular symptoms are well described, cutaneous manifestations of TOS are less often described. We present the case of a 43- year-old woman with acute right upper extremity (RUE) pain, swelling, and progressive erythema with venous engorgement. Initial workup included Doppler ultrasound, coagulation studies, and D-dimer to rule out deep vein thrombosis (DVT), which were all negative. CT chest with contrast revealed no evidence of a thrombus in the right subclavian vein or dilated collateral veins. Rheumatology suspected superficial thrombophlebitis, suggested warm compresses, and RUE elevation. Dermatology was subsequently consulted and identified a constellation of cutaneous signs, including palmar erythema, prominent superficial venous distension on the RUE, petechiae, and pitting edema. These findings prompted repeat vascular evaluation, and venous duplex ultrasonography confirmed venous TOS (vTOS), for which surgical management was scheduled. This case highlights the diagnostic importance of dermatologic assessment in systemic vascular disorders. Cutaneous changes such as unilateral erythema, venous engorgement, temperature asymmetry, and petechiae may represent early indicators of vTOS, yet are frequently misinterpreted as infectious, rheumatologic, or thrombotic conditions. Incorporating dermatologic clues with vascular examination and targeted imaging may reduce misdiagnosis and expedite definitive management.

## Introduction

Thoracic outlet syndrome (TOS) describes a constellation of clinical symptoms resulting from neurovascular compression at the thoracic outlet, most commonly involving the brachial plexus and subclavian vessels. Compression of these structures leads to a constellation of symptoms including pain, paresthesias, and weakness of the neck, upper back, and upper extremities. Depending on the site and nature of compression, TOS is further subclassified into either neurogenic (nTOS), arterial (aTOS), or venous (vTOS) subtypes. TOS may arise from congenital, traumatic, or postural abnormalities that narrow the thoracic outlet space through soft tissue or bony compression [1]. 

TOS most commonly affects patients between the ages of 20 and 40 years, with vascular forms typically seen in younger adults and neurogenic forms being more prevalent overall. The clinical presentation varies widely depending on the structure involved, contributing to frequent diagnostic challenges. aTOS often presents with symptoms suggestive of intermittent ischemia, including pain, pallor, claudication, paresthesias, and cold upper extremities. vTOS commonly presents with arm swelling, cyanosis, and prominent superficial veins, whereas nTOS presents with neck or trapezius pain, supraclavicular pain, shoulder pain, paresthesias, or upper extremity weakness [2].

The treatment of TOS varies depending on the subtype and is usually aimed at symptom management. nTOS is typically treated with physical therapy and anti-inflammatory agents, whereas vTOS is treated with anticoagulation [3,4]. Surgical treatment can be offered in the setting of neuropathic symptoms or vascular compromise, with either nerve decompression or transection of the first cervical rib [5].

Despite recognition of these subtypes, TOS is frequently misdiagnosed or underdiagnosed due to its heterogeneous presentation and symptom overlap with more common musculoskeletal and neurologic conditions. Diagnostic delays are notable in vTOS, where early findings may be attributed to benign etiologies. While neurological and vascular symptoms are well recognized, cutaneous manifestations remain underemphasized in the literature. Importantly, skin findings such as limb swelling, venous distention, and cyanosis may represent some of the earliest clinical signs of venous obstruction.

Herein, we report a case of the successful diagnosis of a 43-year-old female patient with vTOS based on cutaneous manifestations.

## Case presentation

A 43-year-old right-handed female patient presented to our service with a one-week history of acute right upper extremity (RUE) pain, swelling, and progressive color change. The patient reported severe pain that restricted movement, with accompanying paresthesias and numbness. She denied any history of trauma, recent strenuous activity, or heavy lifting. The patient reported no recent exercise, cleaning activity, or repetitive overhead arm use that provoked symptom onset. Symptoms occurred spontaneously without a clear inciting mechanical or postural trigger. Occupational history was not clearly contributory based on available documentation. She noted that the affected extremity became increasingly erythematous throughout the week, with visible venous engorgement. She denied any personal or family history of thrombosis or coagulation disorders. 

The patient was subsequently hospitalized by internal medicine for concern of vascular compromise or cellulitis, and vascular surgery was consulted. Doppler ultrasound, coagulation studies, and D-dimer were ordered to rule out deep vein thrombosis (DVT), which were all negative. CT chest with contrast revealed no evidence of a thrombus in the right subclavian vein or dilated collateral veins. Rheumatology was consulted, who suggested superficial thrombophlebitis and recommended warm compresses and RUE elevation.

**Figure 1:**
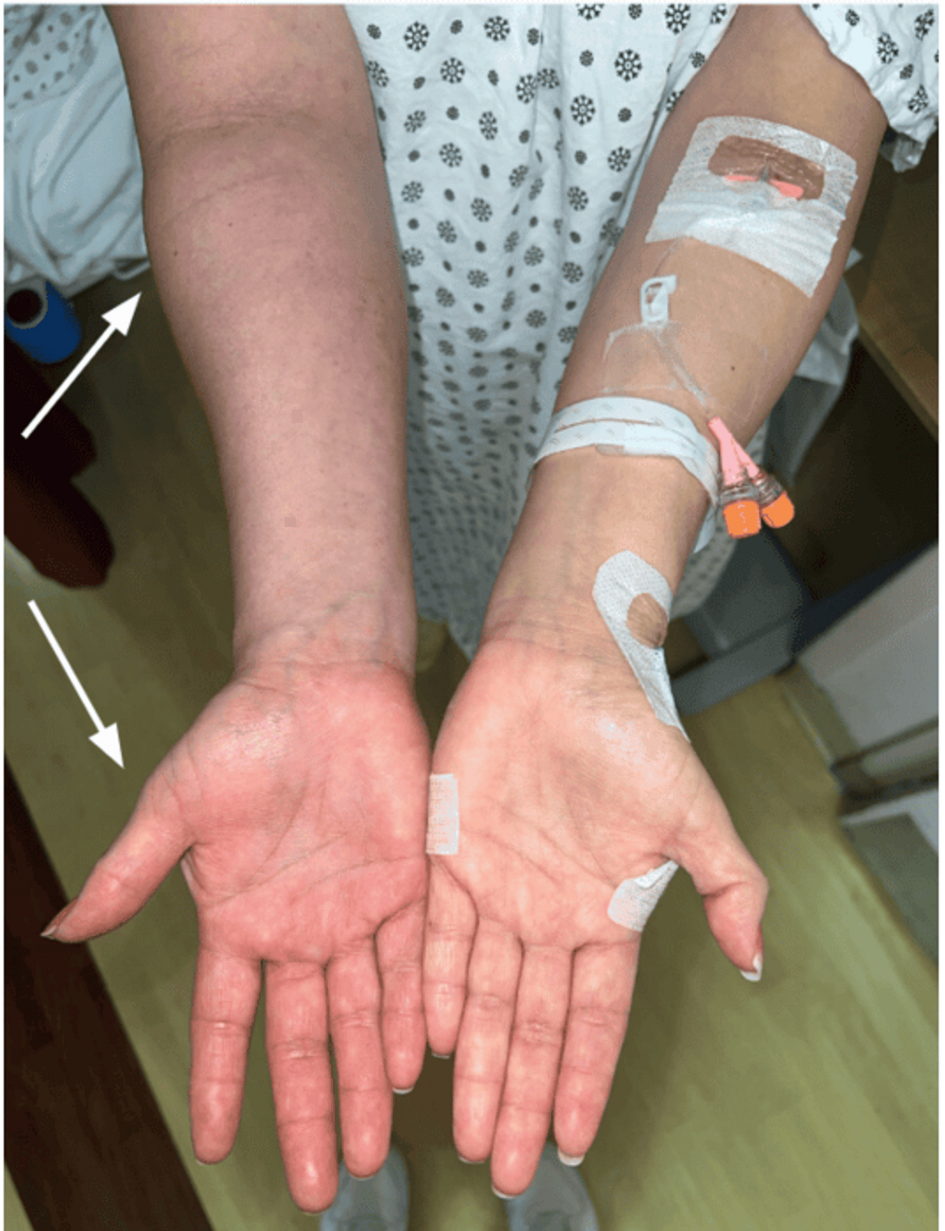
Asymmetric erythema and edema of the right upper extremity compared with the contralateral limb. Arrows highlight areas of prominent swelling and palmar erythema consistent with venous congestion.

**Figure 2:**
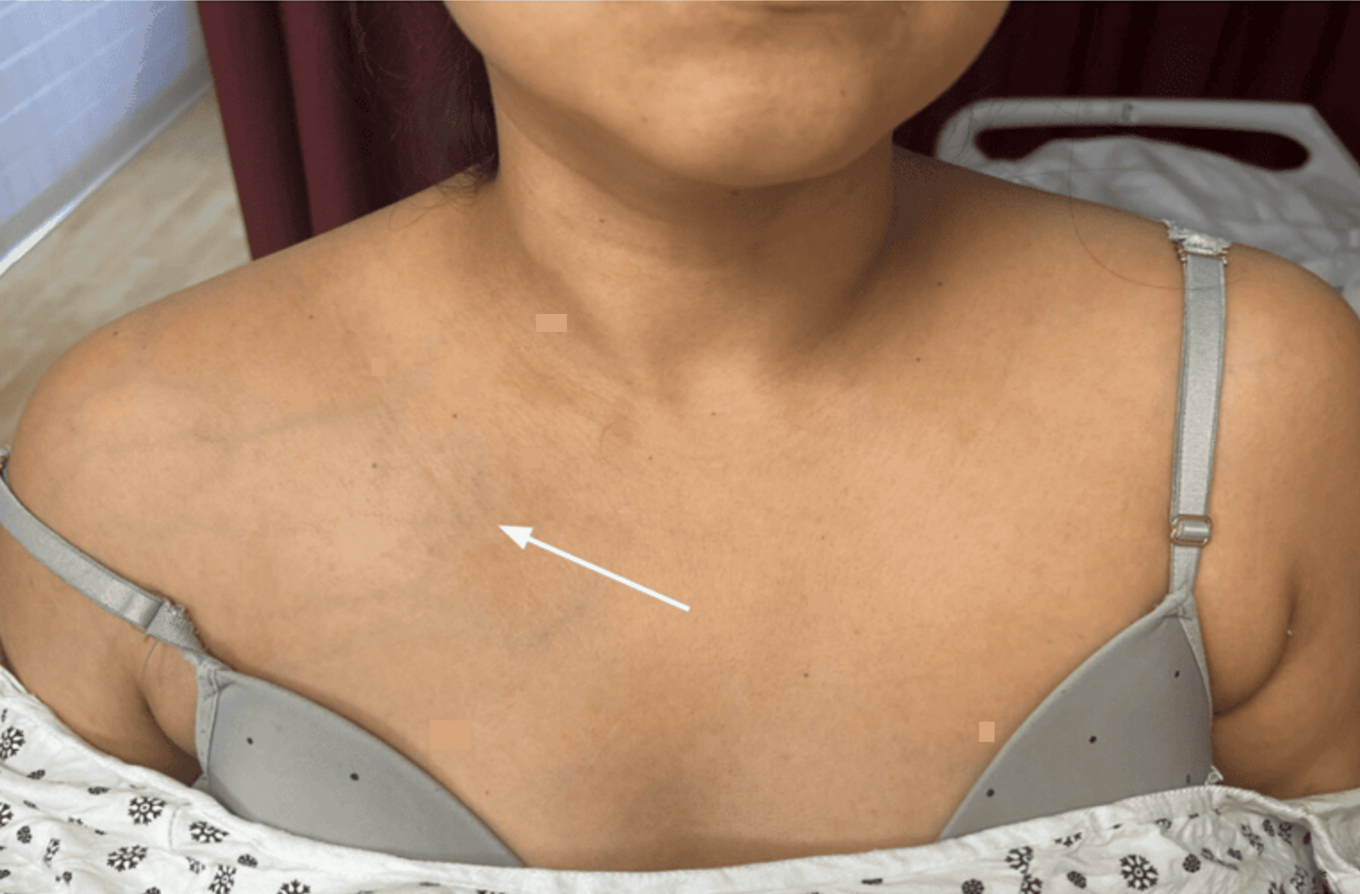
Prominent superficial venous engorgement and erythema extending from the right upper extremity to the anterior shoulder and lateral chest wall. Arrows indicate distended superficial veins and areas of cutaneous vascular congestion.

Following a little symptomatic improvement, dermatology was consulted. Formal provocative maneuvers for TOS, including Adson and elevated arm stress testing, were not documented prior to dermatology evaluation. On our exam, there was a discrepancy in the circumference of right and left upper extremities (right > left), with the right side notably colder in temperature (Figure 1). Radial and ulnar pulses were intact on the right proximal wrist. There was marked palmar erythema and venous engorgement on the RUE extending to the lateral sternal border and right anterior shoulder (Figure 2). Petechiae and pitting edema were also present, and tenderness was elicited upon passive movements of the right elbow, wrist, and shoulder joints. 

Laboratory findings were notable for mild anemia (hemoglobin 10.2 g/dL, hematocrit 30.7%), mild hyponatremia (sodium 133 mmol/L), and low bicarbonate (CO₂) (18 mmol/L) relative to the hospital laboratory's standard reference range (Table 1). Mild anemia was not felt to be contributory to the acute presentation and was considered likely chronic/incidental. Electrolyte abnormalities, including low bicarbonate, were not clinically significant in the context of this patient’s presentation. Coagulation studies and D-dimer were re-ordered.

**Table 1:** Laboratory test results CO2: carbon dioxide

Parameters	Patient Values	Reference Ranges
Hemoglobin (g/dL)	10.2	12.0-15.0
Hematocrit (%)	30.7	36.0-47.0
Mean Corpuscular Volume (fL)	79.9	80-100
Sodium (mmol/L)	133	135-145
CO2 (mmol/L)	18	22-29

Over the course of hospitalization, initial vascular and rheumatologic evaluations remained unrevealing, and symptoms persisted despite conservative management. A repeat vascular surgery consultation was initiated with our diagnosis of vTOS, based on dermatologic findings consistent with venous congestion and subacute vascular compression. Subsequent interval RUE venous duplex ultrasound confirmed the diagnosis, and surgical intervention was scheduled. Although detailed duplex flow measurements and quantitative obstruction severity were not consistently documented in the available records, repeat vascular imaging demonstrated findings consistent with vTOS in the appropriate clinical context.

## Discussion

TOS is a rare yet potentially serious syndrome with significant morbidity when not diagnosed and treated promptly. While neurologic and vascular symptoms are well-recognized in TOS, cutaneous manifestations are infrequently described in the literature. However, changes in skin temperature, color, texture, blotching, hair growth patterns, or sweat production may be observed and should be carefully monitored [6]. Venous-related skin findings such as erythema, petechiae, or superficial vein engorgement are often underreported and may be misinterpreted as infectious or rheumatologic in origin, as was observed in this case [7]. Moreover, acrocyanosis, coolness, and bluish discoloration of the hands, feet, ears, nose, lips, and nipples have been associated with TOS [8]. In rare cases, livedo reticularis has been reported with chronic vascular compression [9]. These dermatologic signs may serve as important diagnostic clues and should raise clinical suspicion for TOS when other common mimickers, such as cellulitis or DVT, have been ruled out.

TOS is often misdiagnosed due to its overlapping symptoms with other disorders such as Raynaud’s syndrome, vasculitis, rotator cuff tear, DVT, or cellulitis [10]. Common risk factors for TOS include repetitive overhead activity, occupational strain, postural abnormalities, and anatomic variants such as cervical ribs or fibrous bands, all of which may contribute to progressive neurovascular compression. Notably, symptoms in the present case occurred without a clear provoking mechanical or occupational trigger, highlighting that vTOS may present spontaneously and without classic risk factors.

Diagnosing TOS can be difficult, and it is often a diagnosis of exclusion. In this case, detailed quantitative vascular measurements such as formal flow velocities or obstruction severity were not consistently documented in early evaluations, which may contribute to diagnostic delay in similar presentations. However, there are established physical examination maneuvers that physicians can elicit to guide diagnosis. Tests such as the Adson's test, Elevated Arm Stress Test, and Upper Limb Tension Tests are designed to provoke vascular or neurologic symptoms by compressing the subclavian vessels or brachial plexus [10]. These maneuvers should be incorporated into physical exams, especially when dermatologic signs are present that raise concern for vascular compromise, as was the case with our patient. Moreover, imaging such as CT or MRI should be performed to evaluate for structural anomalies, such as prominent cervical ribs, fractures, or compressive masses, which can guide both diagnosis and surgical management.

By combining cutaneous findings with these diagnostic strategies, clinicians can more effectively identify TOS, reducing the risk of misdiagnosis and improving patient outcomes.

## Conclusions

This case underscores the diagnostic value of dermatology in identifying cutaneous manifestations of underlying vascular disease. Practicing dermatologists should remain vigilant for asymmetric vascular cutaneous findings, including unilateral skin discoloration, edema, and superficial venous engorgement, which may represent early signs of vTOS even when initial vascular studies are unrevealing. Recognition of these dermatologic clues can prompt timely re-evaluation and facilitate earlier diagnosis and management, ultimately reducing morbidity associated with delayed treatment.
